# High-speed imaging of ultrasonic emulsification using a water-gallium system

**DOI:** 10.1016/j.ultsonch.2020.105387

**Published:** 2020-11-13

**Authors:** Takuya Yamamoto, Ryo Matsutaka, Sergey V. Komarov

**Affiliations:** a Graduate School of Environmental Studies, Tohoku University, Miyagi 980-8579, Japan; b Department of Metallurgy, Tohoku University, Miyagi 980-8579, Japan

**Keywords:** Acoustic cavitation, High-speed imaging, High-speed liquid jet, Ultrasonic emulsification, Droplet and bubble behavior

## Abstract

Aiming at elucidating ultrasonic emulsification mechanisms, the interaction between a single or multiple acoustic cavitation bubbles and gallium droplet interface was investigated using an high-speed imaging technique. To our best knowledge, the moment of emulsification and formation of fine droplets during ultrasound irradiation were observed for the first time. It was found that the detachment of fine gallium droplets occurs from the water-gallium interface during collapse of big cavitation bubbles. The results suggest that the maximum size of cavitation bubble before collapsing is of prime importance for emulsification phenomena. Previous numerical simulation revealed that the collapse of big cavitation bubble is followed by generation of high-velocity liquid jet directed toward the water-gallium interface. Such a jet is assumed to be the prime cause of liquid emulsification. The distance between cavitation bubbles and water-gallium interface was found to slightly affect the emulsification onset. The droplet fragmentation conditions are also discussed in terms of the balance between (1) interfacial and kinetic energies and (2) dynamic and Laplace pressure during droplet formation.

## Highlights

• An emulsification process of water-gallium system during ultrasound irradiation was investigated.
• Formation of ultrafine droplets occurs when a greatly expanded cavitation collapses near the water-gallium interface and the distance between the droplet and bubble is small.
• A high-velocity liquid jet makes a cavity in the surface of gallium forcing it to take a crown shape that eventually results in the droplet fragmentation.
• The above mechanism is supported by a simplified model of droplet formation considering the balance of energy and pressure.

## 1 Introduction

Irradiation of ultrasound waves into liquid causes several phenomena originated from acoustic cavitation. Acoustic cavitation occurs due to sound pressure variation. When the sound pressure reaches sufficiently low values during a rarefaction half-cycle, a small bubble is nucleated and then oscillated non-linearly due to the ultrasound pressure variation, and eventually collapsed (Rayleigh collapse). The bubble collapse causes a shock wave emission and liquid jet near a wall. These phenomena have been thoroughly considered in the relevant papers [1], [2] and book [3]. Based on these phenomena, many applications have been proposed such as ultrasonic cleaning [4], particle separation [5], dispersion [6], emulsification [7], and atomization [8]. In the present study, we focus on the dynamic behaviors of bubble and droplet during ultrasonic emulsification.

Ultrasonic emulsification is one of the applications using ultrasonic waves. When the ultrasound is irradiated onto the interface between two immiscible liquids, emulsion can be rapidly produced. The ultrasonic emulsification is more energetically effective than the other methods such as colloid mill, mechanical agitation and sharpened edge valve [7], [9]. Therefore, the ultrasonic emulsification is widely used for many purposes in food and chemical processes. The ultrasonic emulsification has been studied for many years and results of these studies have been already reviewed [7], [10]. The size of emulsified droplets is decreased with the ultrasonic input power [11]. The effects of ultrasonic frequency, surfactants and volume fraction of dispersion phase on the emulsification efficiency have been also investigated [7], [12]. In these studies, the main focus was placed on the diameter distribution of emulsified droplets that determines the emulsion properties. In addition, ultrasonic emulsification has been practically applied to such fields as pharmacy [13], diesel oil [14], food [15], [16], and etc.

On the other hand, the microscopic phenomena occurring during emulsification are difficult to observe directly. That is why the relationship between acoustic cavitation and emulsification has not been well understood yet. As explained above, ultrasonic irradiation into a liquid causes several phenomena occurring simultaneously that include acoustic cavitation, cavitation bubble oscillation, liquid jetting, macroscopic and microscopic acoustic streaming. To the author’s best knowledge, Li and Fogler were the first who proposed an emulsification mechanism [17]. They suggested that an emulsification proceeds in two steps: droplet fragmentations due to capillary wave and then dispersion due to acoustic cavitation. Later, Kaci *et al*. (2014) investigated an emulsification mechanism when a high-frequency ultrasound was irradiated into distilled water and commercial sunflower oil [18]. In the case of high-frequency ultrasound emulsification, the interfacial instability of droplet oil–water was found to be the dominant factor to proceed emulsification. On the contrary, Zhao *et al*. (2018) investigated an emulsification mechanism using low-frequency ultrasound waves [19]. They proposed that the emulsification takes place in the following two steps: 1. cavitation bubble is covered by oil layer, 2. fine droplets are created at the interface between cavitation bubble and oil layer. Perdish *et al*. (2019) observed an emulsification process through a high-speed camera imaging [20]. They proposed a complicated mechanism driven by liquid jetting and Rayleigh Taylor instability. Yamamoto and Komarov (2020) investigated a dynamic behavior and interaction between cavitation bubbles and droplets through numerical simulation [21]. In this study, the liquid jetting was proposed to be the main factor of emulsification. It was shown that the liquid jet direction is dependent on the droplet characteristics. Besides, the direct interaction between a cavitation bubble and droplet has been clarified for the first time. Later, Orthaber *et al*. (2020) investigated the interaction between a cavitation bubble and an oil droplet using focused laser beams [22]. They also observed a liquid jet from the surface of oil droplet.

As mentioned above, the interaction between liquid jet and droplet surface was found to be of significantly importance for emulsification. However, micro-scale phenomena during emulsification have not been clarified experimentally yet, especially in the case of acoustic cavitation. To shed light on the mechanism of interaction between a cavitation-induced liquid jet and the surface of droplet during emulsification, we carried out an investigation using high-speed imaging in a gallium-water system. The experimental results are discussed based on the energy and pressure balance between the liquid jetting and interfacial force using the results of numerical simulation reported in our previous study [21].

## 2 Experimental

**Fig. 1:**
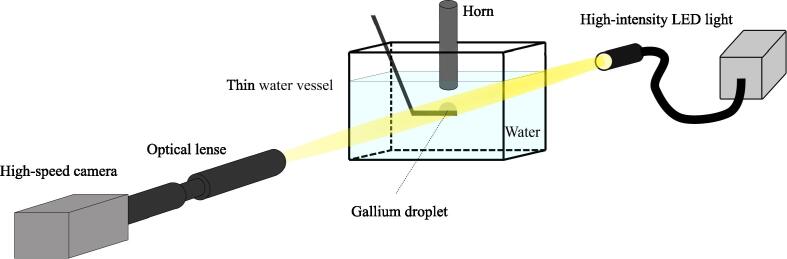
Schematic diagram of experimental apparatus.

In the present study, the dynamic behaviors of cavitation bubble and droplet during emulsification were directly observed with the help of a high-speed imaging technique. Fig. 1 shows a schematic diagram of the experimental apparatus. The gallium droplet was placed on a plate in a shallow water vessel having a width of 10 mm. The temperature of water was set to 40 Celsius degree, which is slightly higher than the melting point of gallium. The distance between the droplet and horn tip was set to 10 mm. The diameter of a titanium horn tip used was 7 mm, and the output power was 84 W, which was measured by power meter. The frequency of ultrasound oscillation was set to 18.35 kHz, which is a resonance frequency for the ultrasound installation used in this study. A high-intensity LED light device (UFLS-751-08W-UT, U-Technology) was used to illuminate the acrylic vessel near the top surface of gallium droplet. A high-speed camera (FASTCAM Nova S12, Photron) was placed in the opposite side of the LED device. An optical lense (LEICA Z16 APO, Leica) was installed in front of the high-speed camera operated at a frame rate of 200,000 fps and a shutter speed of 0.5 μs. Therefore, approximately 11 photographs could be taken during one ultrasonic oscillation cycle. The size of video images was approximately 550 μm in width and 280 μm in height. The pixel resolution of image was 256 × 128. Hence, dimension of each pixel is approximately 2 μm.

The experiments were carried out according to the following procedure.

• Water was filled in the vessel and one droplet of gallium was placed on a solid plate.
• The high-speed camera was switched on to record the water-droplet interface.
• The ultrasound irradiation was started to initiate the gallium droplet emulsification.

## 3 Results and discussion

### 3.1 Observation on dynamic behavior of cavitation bubble during emulsification

**Fig. 2:**
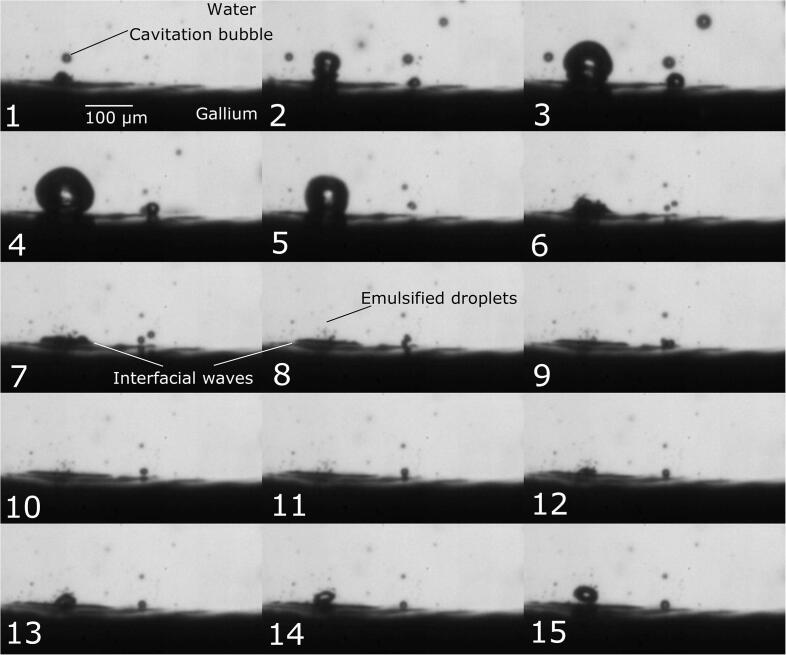
Snapshots of dynamic behaviors of cavitation bubbles and the gallium-water interface during emulsification: The time interval between pictures is 5 μs (A part of supplemental video 1).

**Fig. 3:**
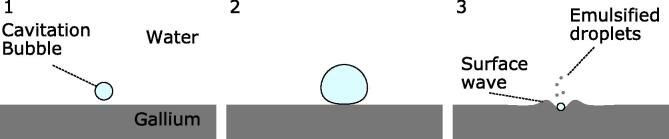
Schematic diagram of dynamic behavior of a cavitation bubble and gallium droplet surface.

First, the dynamic behavior of cavitation bubble during emulsification will be considered. Figure 2 shows snapshots of the dynamic behaviors of a cavitation bubble and the gallium-water interface during emulsification. Figure 3 is a schematic diagram of the emulsification process. More details on the bubble behavior can be seen in the supplementary video 1. The upper grey part of Fig. 2 indicates the water phase while the bottom black part indicates the gallium phase, and the expanding and shrinking circles indicate cavitation bubbles. Initially, the diameter of a large cavitation bubble, indicated in the frame 1 of Fig. 2 by arrow, is approximately 10 μm, and it grows up to approximately 100 μm as seen in frame 4. In this figure, a small interface convexity below the cavitation bubble, indicated by the arrow, is a cavitation bubble adhered onto the interface. After the expansion phase, the bubble shrinks quickly toward the water-gallium interface and collides with it. This results in generation of the interfacial waves at the water-gallium interface as seen in frames 7 and 8. After that (frames 7–11), many tiny gallium droplets are detached from the gallium-water interface. The size of these tiny droplets is smaller than 5 μm, although it is difficult to measure precisely under the given image resolution. Finally, the cavitation bubble is expanded again. These data reveal the way in which the emulsification proceeds in the water-gallium system. The supplementary video 2 gives a clearer understanding of the emulsification mechanism.

**Fig. 4:**
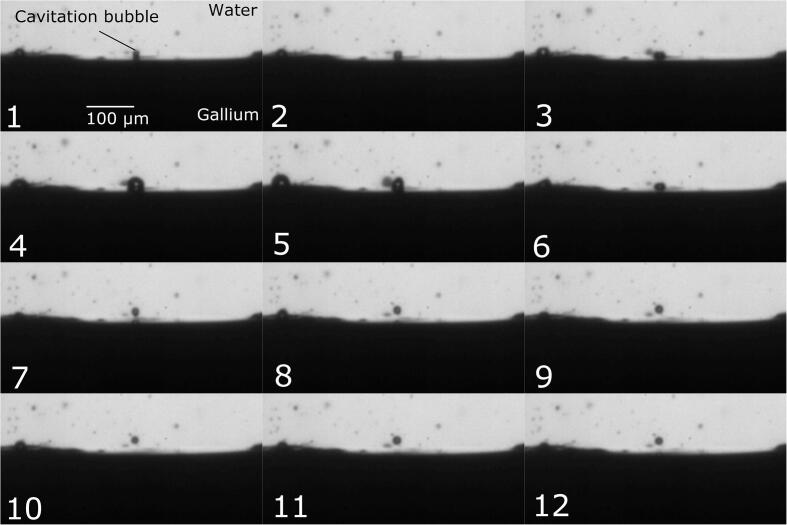
Snapshots of dynamic behaviors of a cavitation bubble and gallium-water interface when emulsification does not occur: The time interval between pictures is 5 μs (A part of supplemental video 1).

Figure 4 shows the dynamic behavior of a cavitation bubble near the gallium-water interface when emulsification does not occur. The cavitation bubble just oscillates non-linearly near the gallium-water interface, and its shape sometimes becomes non-spherical. Notice that the maximum diameter of cavitation bubble is smaller compared with that of previous emulsified cases. Therefore, the size of cavitation bubble appears to be an important factor in determining the emulsification onset. To evaluate the emulsification condition quantitatively, the dynamic behavior of cavitation bubble is discussed in the next section.

### 3.2 Emulsification onset criteria

**Fig. 5:**
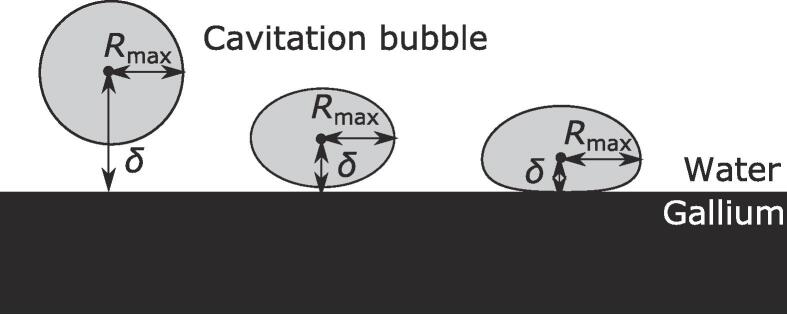
Schematic diagram of definition of the bubble radius, *R_max_* and the distance between the cavitation bubble center and the gallium-water interface, *δ*.

The emulsification onset criteria are evaluated in terms of the maximum radius of cavitation bubble, *R*_max_, and the distance between the water-gallium interface and the cavitation bubble center, *δ*. The definitions of *R*_max_ and δ are shown in Fig. 5. *R_max_* is defined as the maximum radius at a horizontal location, and the bubble center was set at this location. 95 clearly visible images were used to evaluate the emulsification condition.

**Fig. 6:**
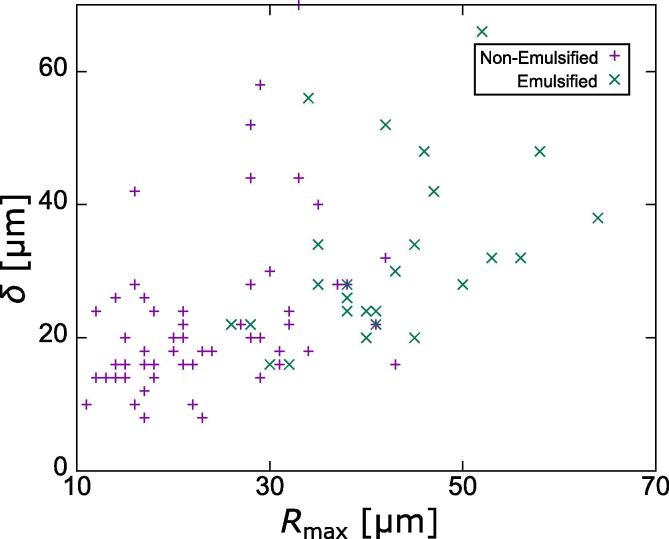
Emulsification map in terms of the maximum bubble radius, *R*_max_ and distance between the cavitation bubble and gallium-water interface, *δ*.

Figure 6 shows the emulsification map in terms of *R*_max_ and δ. The data indicates that there is a relationship between the maximum radius of cavitation bubble and emulsification event. When the maximum radius of cavitation bubble becomes large, emulsification occurs, and vice versa. The distance *δ* seems to contribute the emulsification occurrence. In the case of small distance *δ*, the emulsification occurs easily. The mechanism will be discussed in the next section.

### 3.3 Discussion

**Fig. 7:**
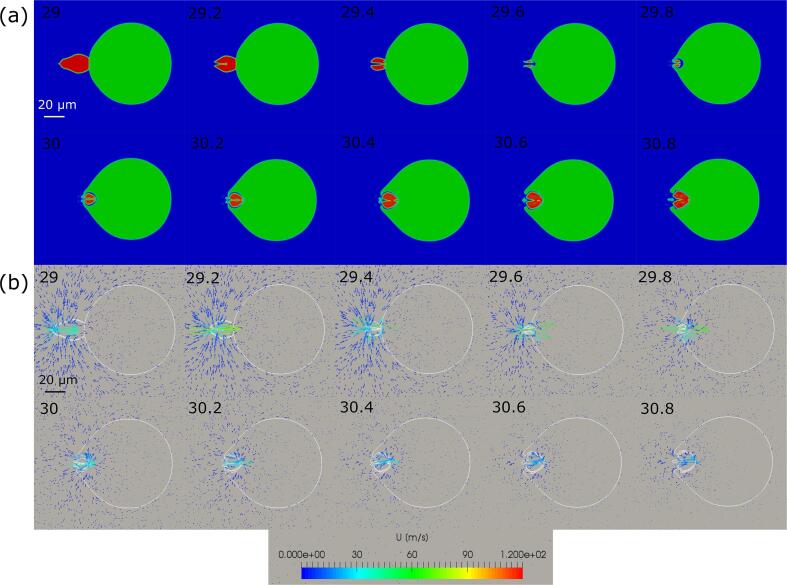
(a) Dynamic behavior of a cavitation bubble and gallium droplet, and (b) time variation in velocity vectors around them: The numbers indicate the elapsed time of sinusoidal pressure oscillation in μsec.

As shown in the previous sections, the emulsification begins after collision of a cavitation bubble with the gallium-water interface during contraction phase. Although we could not observe the dynamic behavior of cavitation bubble just before the collision, the results of our previous study [21] have shown that the generation of liquid jet is the main reason of emulsification. Fig. 7 shows the transient dynamic behavior of a cavitation bubble and gallium droplet, and the velocity vectors around them. The blue, green and red parts indicate the water, gallium and air phases, respectively. The liquid jet occurs and moves toward the gallium droplet creating a cavity on its surface. The edge of gallium droplet takes a crowned shape due to the impact of liquid jet. Finally, the gallium droplet is fragmented producing an ultrafine gallium droplet.

The numerical results indicate that the liquid jet is the key factor to cause the emulsification (ultrafine droplet fragmentation). One can suppose that a fast liquid jet can create a large number of ultrafine droplets. The velocity of such a liquid jet can be evaluated from the expansion ratio of cavitation bubble and distance between the droplet and cavitation bubble. As the expansion ratio increases, the phase shift between the imposed sound pressure and bubble oscillation becomes larger. Obviously, the larger the bubble expansion, the greater is the bubble contraction and the faster is the liquid jet. Therefore, the bubble expansion ratio is an important factor to determine the velocity of liquid jet. As shown in Fig. 6, the emulsification occurs when bubbles are expanded to a size of more than 30 μm. Besides, the jet velocity is dependent on the distance between droplet and cavitation bubble, and the reason for that is as follows. As indicated in our previous study [21], an asymmetric inertial flow around collapsing cavitation bubble plays an important role in controlling the liquid jet velocity. Particularly, it was shown that the larger the asymmetricity of inertial flow, the faster is the velocity of liquid jet. The asymmetricity in turn becomes larger as small distances between the cavitation bubble and droplet.

**Fig. 8:**
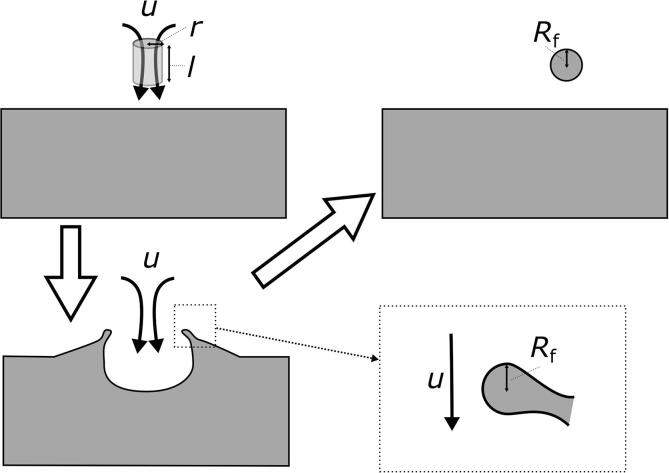
Schematic diagram of fragmentation of a small droplet from big one.

The above results can be additionally discussed on the basis of, first, a balance between the interfacial and kinetic energies, and second, comparison between dynamic and Laplace pressures. Detachment of fine gallium droplets from the interface of a big one is considered to occur as shown in Fig. 8. Two conditions are assumed to be required for the droplet fragmentation due to liquid jet: (1) the kinetic energy of liquid jet must exceed the total interfacial energy of gallium droplets, (2) the dynamic pressure of liquid jet must exceed the Laplace pressure of necked droplet, which is formed before the fragmentation.

In the case shown in Fig. 8, the interfacial energy augmentation after fragmentation, Δ*E_σ_* can be given as:

(1) $$\Delta E_{\sigma }=\sigma \times 4\pi R_{f}^{2}n$$

where *R*_f_ is the radius of fragmented droplet, *n* is the number of fragmented droplet and *σ* is the surface tension. This equation is valid for the case when the radius of all droplets is the same. Assuming that the liquid jet is completely dissipated after the interaction with the water-gallium interface, the reduction in its kinetic energy after the fragmentation, Δ*E*_KE_ can be calculated as

(2) $${\Delta E}_{\mathrm{KE}}=\frac{1}{2}\rho u^{2}V=\frac{1}{2}\rho u^{2}\pi r^{2}l$$

where ρ is the water density, *u* is the liquid jet velocity, *V* is the volume of liquid jet, *r* is the radius of liquid jet, and *l* is the length of liquid jet. The droplet fragmentation takes place when the kinetic energy exceeds the interfacial energy according to Eq. (3):

(3) $$E_{\mathrm{KE}}>E_{\sigma }$$

$$\frac{1}{2}\rho u^{2}\pi r^{2}l>4\pi \sigma R_{f}^{2}n$$

After rearrangement, one can obtain the following relationship between the fragmented droplet radius, *R*_f_ and liquid jet velocity, *u*.

(4) $$R_{f}<ur\sqrt{\frac{\rho l}{8\sigma n}}$$

**Fig. 9:**
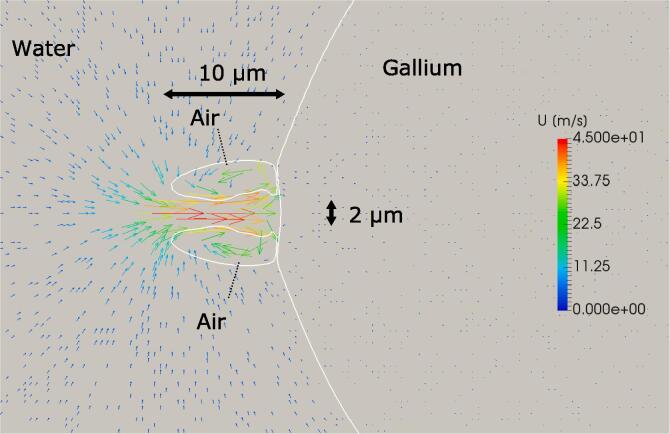
Numerical prediction of a high velocity liquid jet occurrence near the interface between water and big gallium droplet.

Based on the results of our previous studies (Yamamoto and Komarov, 2020), we estimated the liquid jet radius, *r* and length, *l*, to be 1 μm and 10 μm as shown in Fig. 9. By substituting these values and physical properties of liquids in Eq. (4), the following equation can be obtained.

(5) $$R_{f}<4.420\times 10^{-8}un^{-0.5}$$

This equation describes the condition for droplet fragmentation onset.

Next, let’s compare the Laplace pressure and dynamic pressure. The Laplace pressure, *p*_σ_ of a spherical droplet can be given as:

(6) $$p_{\sigma }=\frac{2\sigma }{R_{f}}$$

The dynamic pressure of liquid jet, *p*_KE_ can be expressed in terms of the liquid density and velocity

(7) $$p_{\mathrm{KE}}=\frac{1}{2}\rho u^{2}$$

Based on the above assumption that the droplet fragmentation takes place when the local dynamic pressure exceeds the Laplace pressure of neck-shaped droplet, the condition for formation of a droplet can be given as

(8) $$p_{\mathrm{KE}}>p_{\sigma }$$

Here, we neglected the electrostatic energy because the electrostatic energy is relatively small in micro- or submicro-sized bubbles [23], [24]. By substituting Eqs. (6), (7) into Eq. (8), one can obtain after some rearrangement the following relationship between the fragmented droplet radius, *R*_f_ and liquid jet velocity, *u*.

(9) $$R_{f}>\frac{4\sigma }{\rho }\frac{1}{u^{2}}$$

Substituting the physical properties of liquids in this relationship, one can obtain the following expression

(10) $$R_{f}>2.56\times 10^{-3}\frac{1}{u^{2}}$$

**Fig. 10:**
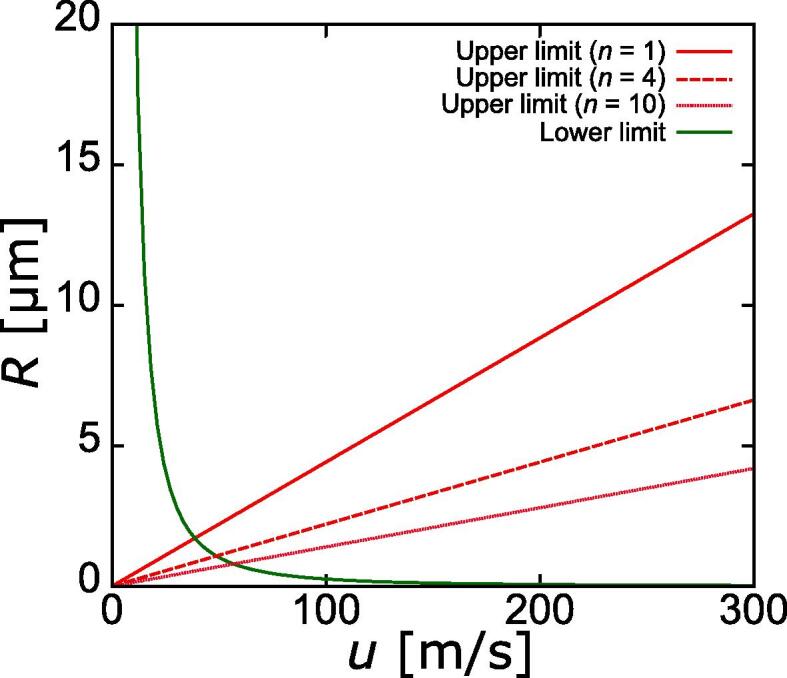
A droplet fragmentation diagram in terms of liquid jet velocity and droplet radius.

Based on the above consideration, a droplet fragmentation diagram can be represented as shown in Fig. 10. The area between one of the energy balance straight lines and the presser balance curve indicates conditions at which the droplet fragmentation becomes possible. This area is indicated as the hatched region for n = 10. It is seen that the minimal velocity of liquid jet required for the fragmentation of one droplet equals 40 m/s under the given conditions. According to the results of our previous study [21], this velocity corresponds to the sound pressure amplitude equal to 0.8 atm. Although the accuracy of the above analysis is rather rough, it allows us to approximately predict the emulsification conditions. Also, Fig. 10 shows that the liquid jet of a higher velocity can generate large and/or small droplets simultaneously. This prediction is in good agreement with the experimental observations recorded in the supplemental video. It is to be noted that the above simplified model calculation is based on the assumption that a single droplet is detached from the mother droplet. However, The above figures and video reveal that a lot of droplets are generated simultaneously. In this case, the maximum size of fragmented droplet becomes smaller than that of above estimation, and vice versa.

It is interesting to compare discussion points on the interaction between the cavitation bubble and droplet during emulsification reported by the other research groups. A liquid jet generated by a cavitation bubble has been studied near a wall [25], [26], [27], [28], [29], [30], [31] and deformable objects like biological cell and oil droplet [20], [21], [22], [32]. In general, the results of these studies reveal that liquid jet generated is directed toward the surface of a solid or rigid (nondeformable) object. On the other hand, in the case of easily deformable object such as oil droplet, the liquid jet is generated from the object [21], [22], [32]. The direct impact of liquid jet on droplets is shown to cause their emulsification in our present and previous studies [21]. On the other hand, Orthaber *et al*. (2020) found that the droplet fragmentation occurs due to pulling of the droplet caused by an oppositely-directed liquid jet [22]. This discrepancy can be explained by the droplet property. As shown in our previous study [21], the direction of liquid jet is dependent on the droplet property. Orthaber *et al*. (2020) used oil to create a droplet. This is the reason why the emulsification mechanism in the present study is different from that of Orthaber *et al*. (2020).

### 3.4 Emulsification caused by multiple cavitation bubbles

**Fig. 11:**
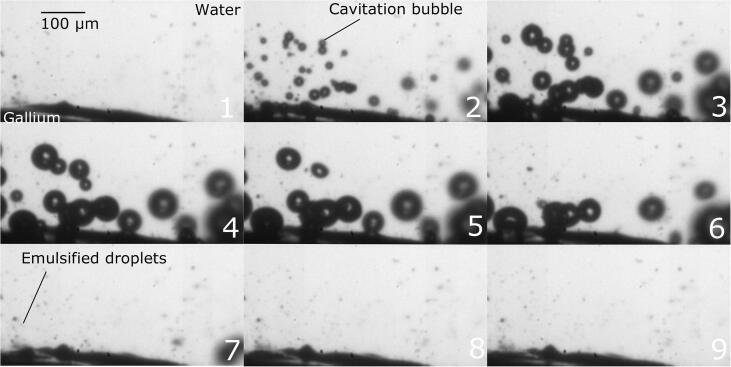
Dynamic behavior of multiple cavitation bubbles during emulsification: The time interval between pictures is 5 μs (A part of supplemental video 3).

The previous section focused on the dynamic behavior of single cavitation bubble near the gallium-water interface. This section presents results on observation of emulsification due to multiple cavitation bubbles. Fig. 11 shows the dynamic behavior of several cavitation bubbles above the water-gallium interface. The first frame corresponds to a moment when the sound pressure is maximum and only few ultrafine bubbles are observable in this picture. Because these ultrafine bubbles are very small in size, it is difficult to distinguish them from gallium droplets in this moment. Then, the fine bubbles begin to expand reaching their maximum size at frames 4 and 5. After that, the bubbles begin to contract as seen from frame 6 and become again invisible at the given resolution in frames 7–9 corresponding to moments of high sound pressure. A lot of tiny gallium droplets are detached from the water-gallium interface during this period as indicated by arrows. Thus, these observations provide a clear picture of emulsification phenomena in a model water-gallium system used in the present investigation. Several cavitation bubbles are observable near the gallium-water interface. The bubbles move toward the gallium-water interface according to the secondary Bjerknes force. This phenomenon enhances the droplet fragmentation due to liquid jet.

## 4 Conclusion

In the present study, an emulsification process of water-gallium system during ultrasound irradiation was investigated. An interaction between acoustic cavitation bubbles and the liquid–liquid interface was clearly observed during ultrasonic emulsification through high-speed camera observations for the first time. Emulsification mechanisms were discussed based on the results of simplified model calculation and numerical simulation of three-phase flow. The results of present study can be summarized as follows:

• Formation of fine droplets occurs when a strongly expanded cavitation bubble collapses near the water-gallium interface and the distance between the droplet and cavitation bubble is relatively small. Emulsification takes place after the cavitation bubble implosion at the gallium-water interface.
• Weakly-expanded cavitation bubbles oscillate spatially in non-linear mode around an equilibrium position without emulsification effect.
• A high-velocity liquid jet makes a cavity in the surface of gallium droplet forcing it to take a crown shape that eventually results in the droplet fragmentation.
• When a cavitation bubble is expanded to a sufficiently large size and located close to a droplet, a strong and fast liquid jet is generated that leads to formation of large number of ultrafine gallium droplets.

The above mechanism is supported by a simplified model of droplet formation considering the balance of energy and pressure.

## CRediT authorship contribution statement

**Takuya Yamamoto:** Writing - original draft, Conceptualization, Methodology, Investigation, Formal analysis, Funding acquisition. **Ryo Matsutaka:** Investigation. **Sergey V. Komarov:** Supervision, Writing - review & editing, Funding acquisition, Project administration.

## Declaration of Competing Interest

The authors declare that they have no known competing financial interests or personal relationships that could have appeared to influence the work reported in this paper.
