# Supplementary figures and images for: High-speed imaging of ultrasonic emulsification using a water-gallium system
Source: Ultrason Sonochem. 2020 Nov 13;71:105387. doi: 10.1016/j.ultsonch.2020.105387 (PMC7786586; doi:10.1016/j.ultsonch.2020.105387)

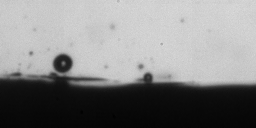

Supplement: Supplementary video 1 [file mmc1.gif]

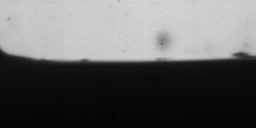

Supplement: Supplementary video 2 [file mmc2.gif]

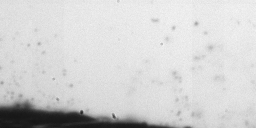

Supplement: Supplementary video 3 [file mmc3.gif]
